# D-dimer Release From Livers During Ex Situ Normothermic Perfusion and After In Situ Normothermic Regional Perfusion: Evidence for Occult Fibrin Burden Associated With Adverse Transplant Outcomes and Cholangiopathy

**DOI:** 10.1097/TP.0000000000004475

**Published:** 2023-05-23

**Authors:** Christopher J.E. Watson, Stephen MacDonald, Christopher Bridgeman, Rebecca Brais, Sara S. Upponi, Theodora Foukaneli, Lisa Swift, Corrina Fear, Linda Selves, Vasilis Kosmoliaptsis, Michael Allison, Rachel Hogg, Kourosh Saeb Parsy, Will Thomas, Rohit Gaurav, Andrew J. Butler

**Affiliations:** 1 Department of Surgery, University of Cambridge, Addenbrooke’s Hospital, Cambridge, United Kingdom.; 2 National Institute for Health and Care Research Cambridge Biomedical Research Centre, Cambridge, United kingdom.; 3 National Institute for Health and Care Research Blood and Transplant Research Unit in Organ Donation and Transplantation, at the University of Cambridge in collaboration with Newcastle University in partnership with National Health Service Blood and Transplant (NHSBT), Cambridge, United Kingdom.; 4 Roy Calne Transplant Unit, Cambridge University Hospitals National Health Service Trust, Cambridge, United Kingdom.; 5 Specialist Haemostasis Laboratory, Cambridge University Hospitals National Health Service Trust, Cambridge, United Kingdom.; 6 Department of Histopathology, Cambridge University Hospitals National Health Service Trust, Cambridge, United Kingdom.; 7 Department of Radiology, Cambridge University Hospitals National Health Service Trust, Cambridge, United Kingdom.; 8 Department of Haematology, Cambridge University Hospitals National Health Service Trust, Cambridge, United Kingdom.; 9 Department of Medicine, Cambridge University Hospitals NHS Trust, Cambridge, United Kingdom.; 10 Statistics and Clinical Research, NHS Blood and Transplant, Bristol, United Kingdom.

## Abstract

Deceased donor livers are prone to biliary complications, which may necessitate retransplantation, and we, and others, have suggested that these complications are because of peribiliary vascular fibrin microthrombi. We sought to determine the prevalence and consequence of occult fibrin within deceased donor livers undergoing normothermic ex situ perfusion (NESLiP) and evaluate a role for fibrinolysis.

**Methods.:**

D-dimer concentrations, products of fibrin degradation, were assayed in the perfusate of 163 livers taken after 2 h of NESLiP, including 91 that were transplanted. These were related to posttransplant outcomes. Five different fibrinolytic protocols during NESLiP using alteplase were evaluated, and the transplant outcomes of these alteplase-treated livers were reviewed.

**Results.:**

Perfusate D-dimer concentrations were lowest in livers recovered using in situ normothermic regional perfusion and highest in alteplase-treated livers. D-dimer release from donation after brain death livers was significantly correlated with the duration of cold ischemia. In non-alteplase-treated livers, Cox proportional hazards regression analysis showed that D-dimer levels were associated with transplant survival (*P* = 0.005). Treatment with alteplase and fresh frozen plasma during NESLiP was associated with significantly more D-dimer release into the perfusate and was not associated with excess bleeding postimplantation; 8 of the 9 treated livers were free of cholangiopathy, whereas the ninth had a proximal duct stricture.

**Conclusions.:**

Fibrin is present in many livers during cold storage and is associated with poor posttransplant outcomes. The amount of D-dimer released after fibrinolytic treatment indicates a significant occult fibrin burden and suggests that fibrinolytic therapy during NESLiP may be a promising therapeutic intervention.

## Graphical abstract

**Figure:**
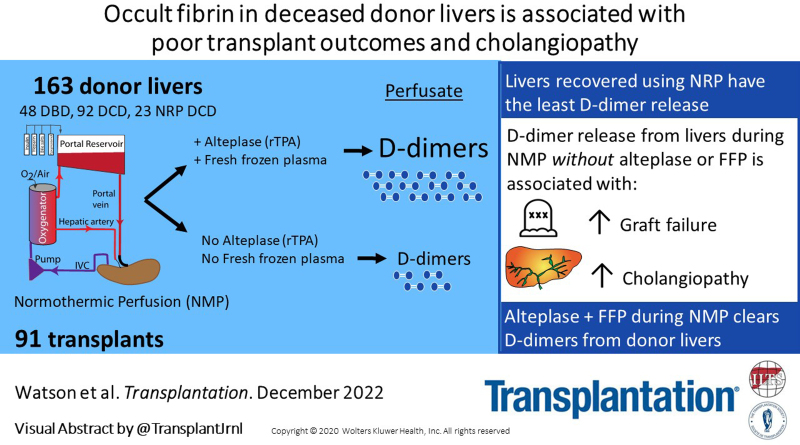

## INTRODUCTION

Livers from deceased donors are susceptible to biliary complications, such as leaks and anastomotic and nonanastomotic strictures (NAS), which may necessitate endoscopic, radiological, or surgical attention, and, in some cases, may require retransplantation. Livers donated after circulatory death (DCD) are particularly prone to such complications and have also been associated with poor graft survival,^1^ although results have improved in recent years following efforts to use grafts with short periods of warm and cold ischemia.^2^ Interventions such as in situ normothermic regional perfusion (NRP), normothermic ex situ liver perfusion (NESLiP), and hypothermic oxygenated perfusion (HOPE) have been reported to achieve improved results compared with DCD livers treated solely by cold storage, both in terms of survival and reduction of biliary complications.^3-6^

The similarity of NAS to the appearance of biliary strictures that follow hepatic artery thrombosis has suggested an ischemic origin, possibly secondary to microvascular thrombi. Accordingly, surgeons have flushed the liver with fibrinolytic agents, such as recombinant tissue plasminogen activator (rTPA) at procurement^7^ or instilled rTPA into the hepatic artery during implantation,^8,9^ and claimed reduction in the incidence of NAS, albeit with a risk of bleeding.

We have reported stromal necrosis in the large intrahepatic bile ducts of some livers that underwent NESLiP^10^ and observed that the areas of necrosis were associated with fibrin microthrombi plugging peribiliary vessels, providing more evidence to support a microvascular cause of NAS.^11^ We also showed that a combination of alteplase rTPA (Actilyse, Boehringer Ingelheim, Germany), given with fresh frozen plasma (FFP) as a source of plasminogen, was associated with an absence of fibrin in the intrahepatic peribiliary vessels, but this was not seen when alteplase was given alone without a plasminogen source. Others have noted microthrombi in the hepatic sinusoids,^12^ an observation that probably explains the improvement in hemodynamics, which has been observed when fibrinolytic therapy is given during NESLiP.^13^

D-dimers are a product of fibrin breakdown. To assess the prevalence of fibrin in deceased donor livers and its consequences, we analyzed the release of D-dimers from livers undergoing NESLiP and correlated this with posttransplant outcomes. We also report some preliminary observations on alteplase therapy during NESLiP.

## MATERIALS AND METHODS

### Liver Perfusion

**FIGURE 1.**
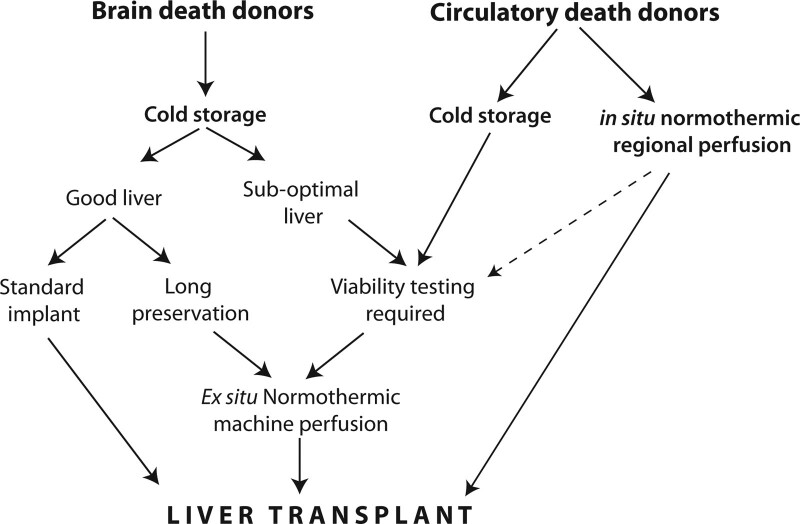
Cambridge protocol for using in situ normothermic regional perfusion and ex situ normothermic liver perfusion. DCD livers were preferentially recovered using NRP, but where that was not possible, DCD livers underwent NESLiP. DBD livers underwent NESLiP if there was doubt about viability or where a long preservation time was envisaged for recipient or logistical reasons. DBD, donation after brain death; DCD, donation after circulatory determined death; NESLiP, normothermic ex situ perfusion; NRP, in situ normothermic regional perfusion.

Livers designated for research having been declined by all national liver transplant units (n = 24) or accepted for clinical use (n = 139) underwent a period of NESLiP. NESLiP was used clinically for livers where there was doubt regarding viability or the ischemic time was likely to be prolonged and latterly for all DCD livers that had not undergone NRP (Figure 1). Livers undergoing NESLiP in this study were perfused using either the Liver Assist (XVIVO, The Netherlands) or the *metra* (OrganOx, United Kingdom), and the technique is detailed in the **Supplemental Digital Content** (**SDC**, http://links.lww.com/TP/C653). Only livers that underwent NESLiP for which consent had been given for research and perfusate samples had been stored for future analysis were included in the study.

Perfusate samples were taken at prespecified time points; the supernatant was immediately separated from the red cell mass by refrigerated centrifugation and then frozen in liquid nitrogen before being transferred to a –80 °C freezer. It is the analysis of these samples, in particular those taken after 2 h of NESLiP, that forms the main part of this report.

### Preperfusion Liver Flush Protocol

Before undergoing NESLiP, all livers were flushed with compound sodium lactate (Hartmann’s solution, Baxter Healthcare Ltd, United Kingdom) at room temperature to de-air the cannulas and warm and preload the liver with lactate. A small sample of vena caval effluent from this preperfusion flush from 20 consecutive livers was also collected for D-dimer analysis to assess whether fibrin was present in livers before NESLiP began.

### Liver Transplantation

A decision to transplant a liver undergoing NESLiP followed our published criteria.^10^ Liver transplantation followed standard surgical practice, as did the use of blood and blood products. Magnetic resonance cholangiopancreatography was undertaken only when indicated clinically. Biliary leaks and anastomotic strictures were defined as those requiring endoscopic, surgical, or radiological treatment. All NAS are reported.

### Alteplase/FFP Regimens

Alteplase was used in 5 different regimens, administered to DCD donor livers either alone or with FFP; given separately either directly into the hepatic artery cannula, where the FFP and alteplase mixed, or into the portal reservoir; and given as either a single dose or a combination of bolus followed by infusion (Table 1). Alteplase was reconstituted according to the manufacturer’s instructions, and infusions commenced at the start of NESLiP. Care was taken to avoid mixing FFP and alteplase for any longer than possible before the infusion commenced.

**TABLE 1.** Fibrinolytic regimens

Alteplase					FFP					
Group	Bolus (mg)	Infusion (mg)	Duration (min)	Delivered into	Bolus (mL)	Infusion volume (mL)	Duration (min)	Delivered into	Number treated	No. of transplants
A	50	0	0	HA	–	–		–	4	1
B	0	20	60	HA	250	0	60	Reservoir	5	0
C	10	40	80	Reservoir	50	200	80	Reservoir	5	4
D	5	45	180	Reservoir	25	225	180	Reservoir	3	1
E	10	40	180	HA	25	225	180	HA	5	3

Note that the volume of FFP has been assumed to be 250 mL but, in reality, varied from 245 to 292 mL. The bolus volume was always as stated in the table. Group A was perfused on the Liver Assist and the other 4 groups on the OrganOx metra. Infusions into the hepatic artery were delivered via a 3-way tap on the arterial cannula; infusions into the blood reservoir were similarly delivered using a 3-way tap on top of the reservoir.

FFP, fresh frozen plasma; HA, hepatic artery.

Groups A and B comprised declined livers accepted for research to evaluate liver histology after treatment with alteplase, although 1 liver with excellent perfusion characteristics was transplanted. Livers in group A were perfused on the XVIVO Liver Assist and the other 4 groups on the OrganOx *metra.* Livers in groups C, D, and E were perfused with alteplase, having been accepted for transplantation.

The intraoperative blood replacement requirements of livers receiving alteplase and FFP were compared with the requirements of a comparator group of our last 18 transplanted livers that had undergone NESLiP without alteplase.

Alteplase-treated livers that failed viability criteria had a 2-cm thick section taken in a transverse plane to include common hepatic duct and its bifurcation. These were stained with hematoxylin and eosin and examined at multiple levels.

### D-dimers

D-dimers were measured using latex immunoturbidimetry with the D-dimer HS assay on the ACLTOP 750CTS analyzer (Werfen, Barcelona, Spain) according to the manufacturer’s instructions (see **Supplemental Digital Content**, **SDC**, http://links.lww.com/TP/C653).

### Approvals

Informed consent for liver perfusion research was obtained from patients or the next-of-kin of donors for livers used for research only, and the protocols conformed to the 1975 Declaration of Helsinki and were approved by UK research ethics committees.

### Statistics

Statistical analysis is detailed in the **Supplemental Digital Content** (**SDC**, http://links.lww.com/TP/C653).

## RESULTS

### Two-hour D-dimer Concentrations of Perfused Livers

**FIGURE 2.**
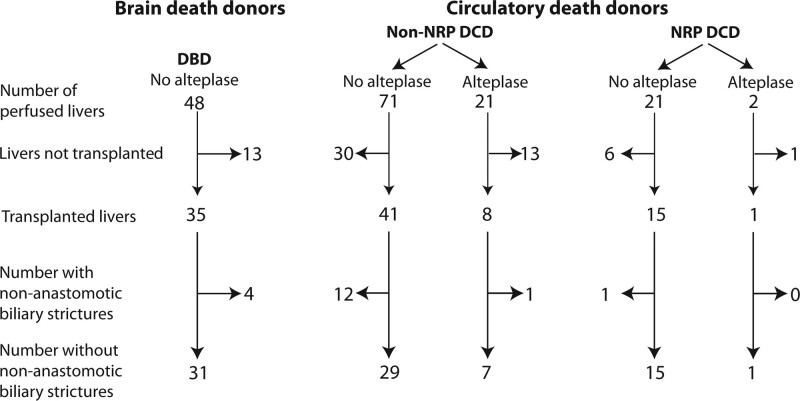
Flow diagram illustrating perfusates studied by donor type and treatment. Samples of perfusate from 163 livers taken after 2 h of NESLiP were analyzed. These represent samples from all the liver perfusions in which ethical approval had been obtained for research where samples existed. DBD, donation after brain death; DCD, donation after circulatory determined death; NESLiP, normothermic ex situ perfusion; NRP, in situ normothermic regional perfusion.

Two-hour perfusate samples were available on 163 livers: 48 donation after brain death (DBD) and 115 DCD livers, of which 23 had also undergone NRP and 23 had been treated with alteplase (Figure 2). Eighteen livers developed NAS. Perfusate samples were not available on 2 alteplase-treated livers.

**FIGURE 3.**
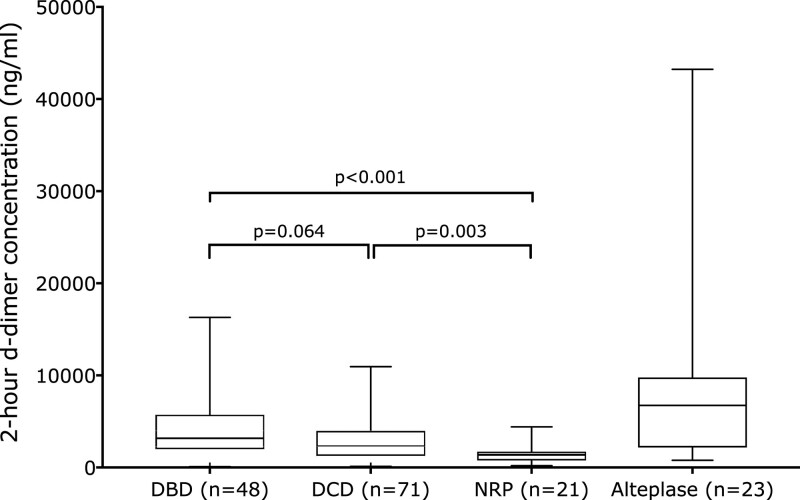
Box and whisker plot of perfusate D-dimer concentrations after 2 h of NESLiP by donor type and treatment. The box represents the interquartile range, the horizontal line the median of the group, and the whiskers the range. There was a significant difference in 2-h D-dimer levels across all groups (Kruskal-Wallis *P* < 0.001), with a difference also when the alteplase-treated DCD livers are ignored (Kruskal-Wallis *P* < 0.001). Statistical significance between groups was estimated by the Kolmogorov-Smirnov test and is indicated on the chart. The alteplase-treated group also had D-dimer levels that were significantly higher than NRP (*P* < 0.001), DCD livers (*P* = 0.008), and DBD livers (*P* = 0.041). DBD, donation after brain death; DCD, donation after circulatory determined death; NESLiP, normothermic ex situ perfusion; NRP, in situ normothermic regional perfusion.

There was no difference in D-dimer levels between DBD and non-NRP DCD livers (*P* = 0.064), but NRP livers had significantly lower D-dimer levels than both DBD (*P* < 0.001) and DCD (*P* = 0.003) livers (Figure 3). Alteplase-treated livers had the highest D-dimer levels, which were significantly higher than nonalteplase-treated DBD (*P* = 0.041), DCD (*P* = 0.008), and NRP livers (*P* < 0.001).

DBD livers that passed viability assessment and were transplanted had significantly lower D-dimer levels than nontransplanted DBD livers (*P* = 0.012), although there was no significant difference in DCD livers (*P* = 0.343) or NRP livers (*P* = 0.880; **Figure S1**, **SDC**, http://links.lww.com/TP/C653). Livers that had undergone NRP and were undergoing NESLiP for logistical or recipient reasons had numerically lower D-dimer levels than NRP livers undergoing NESLiP because of concerns about NRP (*P* = 0.089; **Figure S2**, **SDC**, http://links.lww.com/TP/C653).

There was no difference in 2-h D-dimer levels of livers perfused with human albumin solution (n = 103) or Gelofusine (BBraun, n = 40) without alteplase, with median values of 2318 ng/mL (interquartile range, 1381–4398 ng/mL) for human albumin solution and 2250 ng/mL (interquartile range, 944–4967 ng/mL) for Gelofusine (*P* = 0.670; **Figure S3**, **SDC**, http://links.lww.com/TP/C653).

### D-dimers in Preperfusion Effluent

**FIGURE 4.**
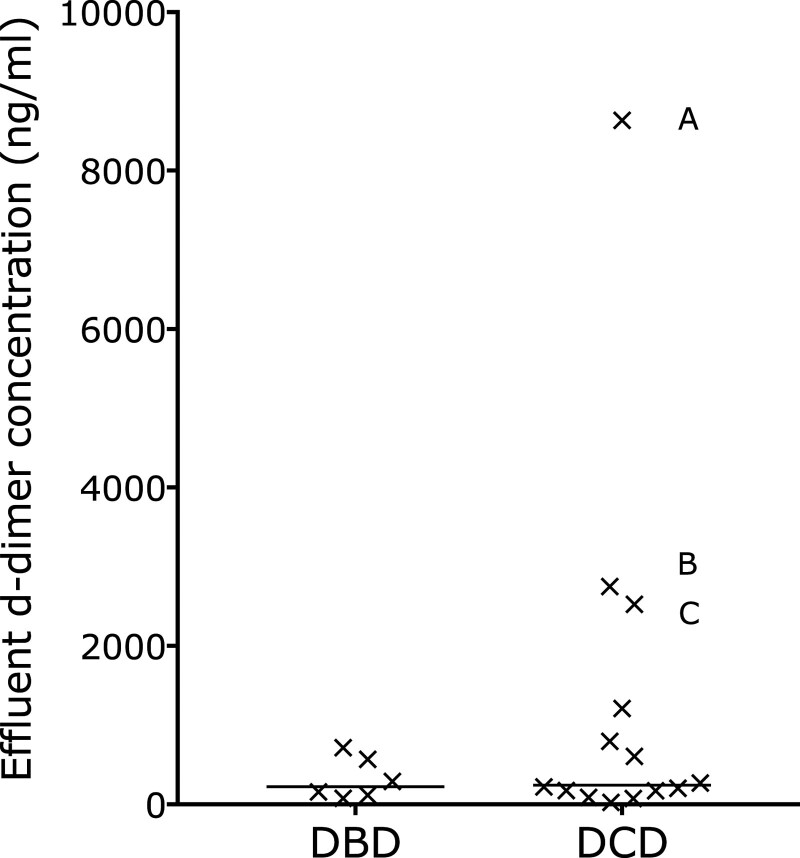
D-dimer concentrations in the preperfusion effluent of the livers. Livers were flushed with 2 L of Hartmannn’s solution before being placed on the perfusion machine, and the effluent from the vena cava was collected and sampled. Seventeen of the 20 livers studied had effluent concentrations of D-dimers over 100 ng/mL. There were 3 DCD livers with particularly high D-dimer concentrations: liver A failed viability testing, liver B suffered primary nonfunction, and liver C developed severe vasoplegia requiring epinephrine and external cardiac massage. DBD, donation after brain death; DCD, donation after circulatory determined death.

To determine whether fibrin was in the liver before it underwent NESLiP, we examined the preperfusion effluent flushed from 20 livers, 6 DBDs, and 14 DCDs: D-dimers were found in all livers, with 17 having levels >100 ng/mL (Figure 4). Three DCD livers had particularly high levels: the highest was in a liver that failed viability criteria, the next highest concentration was seen in a liver that suffered primary nonfunction, and the third highest was in a liver whose recipient suffered profound postreperfusion vasoplegia requiring external cardiac massage and epinephrine support. There was no correlation between effluent D-dimers and 2-h perfusate D-dimers in the 15 effluent cases that had not received alteplase (Spearman *r* = 0.343, *P* = 0.211).

### D-dimer Levels and Cold Ischemia

Because D-dimers were present before NESLiP, we went on to examine whether the duration of cold ischemia affected 2-h perfusate D-dimer levels. There was a positive correlation with cold ischemic time for DBD livers (Spearman *r* = 0.399, *P* = 0.007) but no correlation with NRP or DCD livers (**Figure S4**, **SDC**, http://links.lww.com/TP/C653). Similarly, there was no correlation between 2-h D-dimer levels and the time from withdrawal of treatment to asystole or asystole to in situ perfusion for DCD livers.

### Transplant Outcomes and 2-h D-dimer

**FIGURE 5.**
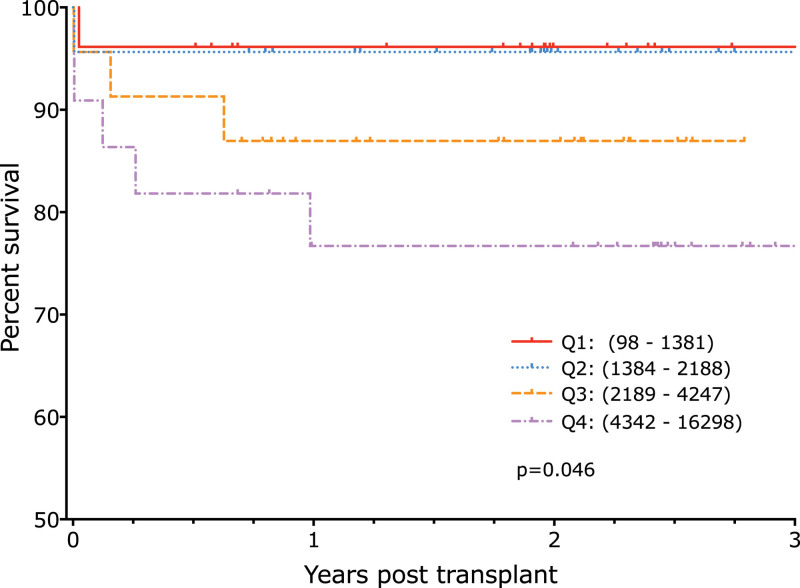
Transplant survival according to 2-h perfusate D-dimer concentrations. Transplant survival (graft survival not censored for death) is worse with increasing D-dimer concentration quartile.

Perfusate samples taken after 2 h of NESLiP were available on 91 transplanted livers. These were divided into quartiles according to increasing D-dimer concentrations. Transplant survival (graft survival not censored for death) was significantly worse with increasing 2-h D-dimer concentration quartiles (Figure 5; *P* = 0.046). There was no significant difference between quartiles in the model for early allograft function score^14^ or in the incidence of acute kidney injury.^15^

**FIGURE 6.**
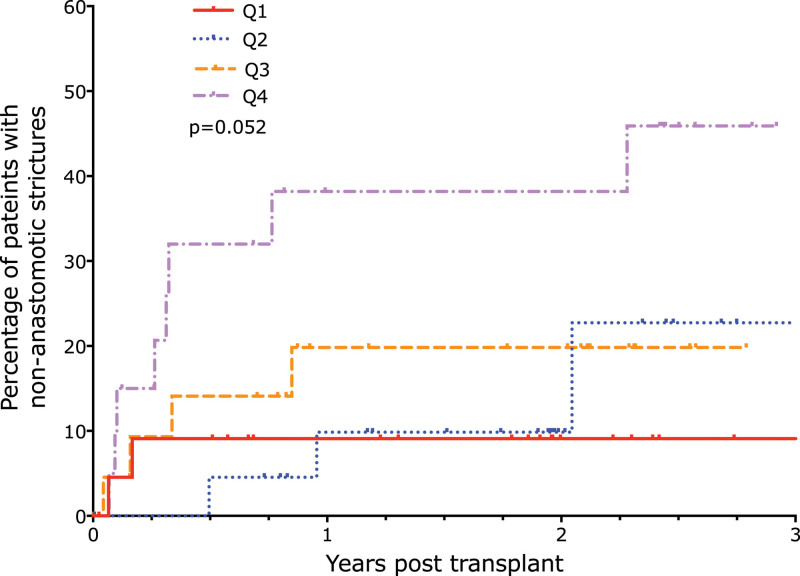
Cumulative incidence of nonanastomotic stricture by 2-h D-dimer quartile. Increasing D-dimer concentration quartiles were associated with an increased incidence of nonanastomotic biliary strictures.

NAS were more common in the quartiles with the highest D-dimer levels (Figure 6; *P* = 0.052). The incidence of anastomotic leaks and anastomotic strictures requiring surgical or radiological intervention was also highest in the quartiles with the greatest D-dimer levels (Table 2).

**TABLE 2.** Biliary complications by 2-h D-dimer quartile in nonalteplase-treated livers

Quartile	2-h D-dimer range (ng/mL)	Nonanastomotic strictures	Anastomotic strictures^*a*^	Anastomotic leaks^*a*^	Total number of livers with any biliary complication
1	98–1381	3	3	0	6
2	1384–2188	2	3	0	5
3	2189–4247	4	8	0	8
4	4342–16 298	8	6	2	11

*^a^*Defined as those requiring surgical, endoscopic, or radiological intervention. Note that some livers may have developed >1 type of complication.

Eight of the 91 patients died during follow-up (**Supplemental Digital Content** and **Table S1**, **SDC**, http://links.lww.com/TP/C653). Two of the deaths were from multiorgan failure in patients with venous outflow obstruction with a significant transhepatic gradient, both in patients with high D-dimer levels. A further 2 deaths were from primary nonfunction (PNF); both of these livers had high levels of D-dimers.

### Regression Analysis of Transplant Survival

To further investigate the association of 2-h D-dimer levels with transplant outcome, the 1-y transplant survival was adjusted using a Cox proportional hazards regression model for donor age, cold ischemia time, and donor type (DBD, DCD, and NRP). D-dimer level was then added to the model and found to be significantly associated with transplant survival (likelihood ratio test *P* value = 0.005), with higher D-dimer levels associated with a higher chance of suffering transplant failure within 1 y (hazard ratio for a 100-unit increase in D-dimer level: 1.028; 95% CI, 1.010-1.047). Although there is some evidence of an interaction between D-dimer level and donor type, because of the low number of transplant failures, there is not enough power to account for this interaction in the Cox analysis.

### Multivariable Analysis of Nonanastomotic Biliary Strictures

The development of NAS within 1 y was adjusted using a Cox proportional hazards regression model for donor age, cold ischemia time, and donor type. D-dimer level was then added to the model and found to be weakly associated with stricture development (likelihood ratio test *P* value = 0.084), with higher D-dimer levels associated with a higher chance of developing strictures within 1 y (hazard ratio for a 100-unit increase in D-dimer level: 1.015; 95% CI, 0.999-1.031).

### Change in D-dimer Concentration Over Time During Treatment With Alteplase

**FIGURE 7.**
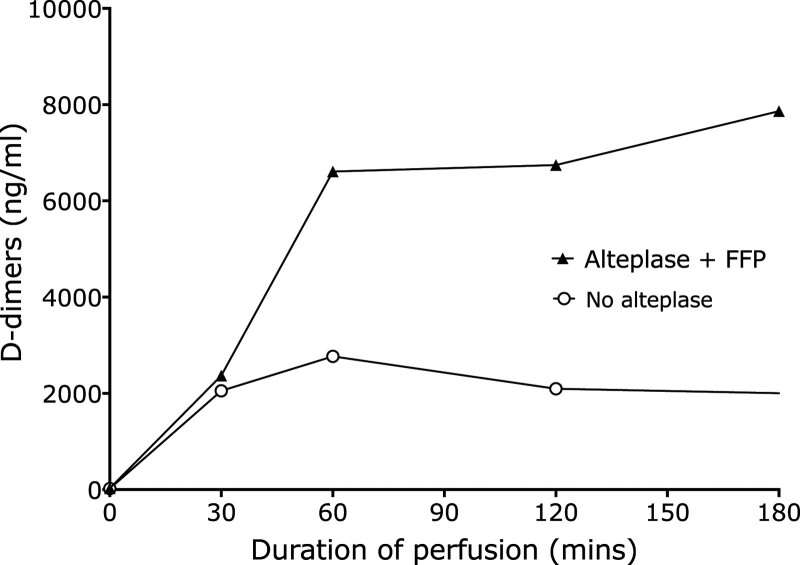
Rate of change of D-dimer concentrations over time with and without alteplase/FFP treatment. D-dimer concentrations (median) in the perfusate of 19 livers that underwent NESLiP and that had received either bolus or an infusion of alteplase with FFP and 8 livers that did not receive alteplase and FFP. FFP, fresh frozen plasma; NESLiP, normothermic ex situ liver perfusion.

To assess the time course of fibrinolysis with alteplase, serial perfusate samples that were available for 19 livers treated with alteplase and FFP were studied. D-dimer concentrations were found to increase in the first hour, after which the median levels plateaued, implying there was no freshly formed fibrin to break down despite the alteplase/FFP continuing for up to 3 h in some treatment groups (Figure 7). These profiles were compared with 7 non-alteplase/FFP livers, which showed a similar early increase in D-dimer release before plateauing at an hour, albeit with lower levels of D-dimer release than the alteplase livers.

### Outcomes of Livers Treated With Alteplase

Nine livers that had undergone alteplase therapy were transplanted. One of the alteplase-treated livers from regimen C developed a hilar biliary stricture, but the 2 alteplase-treated livers with the highest 2-h D-dimers, levels much higher than the highest seen in any nonalteplase transplant quartile, have no clinical features of NAS and have normal liver chemistry at 245 and 469 d (Table 3).

**TABLE 3.** Transplant outcomes

Alteplase regimen	Donor age	US DRI	UK DLI	Withdrawal time (min)	Asystolic time (min)	CIT (min)	NESLiP duration (min)	2-h D-dimers (ng/mL)	MEAF score	MRCP results	Follow-up (d)
A	69	3.6	2.2	41	10	383	448	782	2.5	Not done	>1000
A	44	2.1	2.0	4	8	261	297	8693	Not transplanted		
A	60	2.8	2.2	6	12	456	180	1060	Not transplanted		
A	35	1.7	1.7	7	13	323	163	7345	Not transplanted		
B	54	2.8	1.8	9	12	715	549	3629	Not transplanted		
B	63	3.3	2.7	27	13	531	242	9783	Not transplanted		
B	31	2.0	1.5	16	13	458	373	7902	Not transplanted		
B	50	2. 7	1.5	24	14	527	240	N/A	Not transplanted		
B	68	3.4	2.9	10	11	572	310	6224	Not transplanted		
C	52	2.8	2.5	14	13	652	255	2175	Not transplanted		
C	42	2.2	1.8	9	11	389	514	7376	5.4	Stricture^*a*^	490
C	48	2.6	2.1	11	16	409	459	N/A	3.7	Not done	443
C	30	1.8	1.5	12	9	445	917	27 393	5.7	Not done	469
C	48	2.5	2.4	76	16	480	421	8150	4.8	Normal	446
D	57	2.7	2.8	15	11	447	247	6745	Not transplanted		
D	23	1.9	1.6	10	9	483	262	12 905	Not transplanted		
D	43	2.2	1.9	16	12	434	510	5000	3.1	No MRCP	353
E	50	2.7	2.4	10	19	399	268	11 337	Not transplanted		
E	37	2.1	2.3	16	8	366	369	1330	Not transplanted		
E	30	2.1	1.4	14	11	405	734	2280	3.3	Normal	260
E	51	3.0	1.9	28	10	497	732	43 230	1.3	Normal	301
E	17	1.8	1.5	6	11	319	485	1831	7.1	Not done	222
Tx DBD^*b*^	44 (29–54)	1.6 (0.4–1.9)	1.0 (0.8–1.2)	–		460 (368–587)	491 (355–580)	2383 (1554–4773)	4.2 (3.0-5.5)		
NotTx DBD^*b*^	56 (47–66)	1.9 (1.9–2.4)	1.0 (1.0–1.3)	–		523 (422–724)	316 (237–500)	6262 (4030–7657)	–		
Tx DCD^*b*^	50 (29–56)	2.4 (2.0–2.7)	1.8 (1.6–2.2)	14 (11–16)	12 (10–14)	417 (380–467)	520 (449–655)	2523 (1365–5858)	3.6 (2.6–5.6)		
NotTx DCD^*b*^	54 (43–65)	2.8 (2.3–3.2)	2.0 (1.7–2.3)	14 (8–26)	12 (11–14)	491 (421–627)	336 (241–404)	1734 (796–3892)	–		
Tx NRP^*b*^	46 (35–57)	2.2 (1.9–2.4)	1.9 (1.7–2.3)	16 (12–28)	17 (14–19)	298 (261–389)	469 (430–565)	1381 (720–1673)	4.5 (3.8–7.0)		
NotTx NRP^*b*^	55 (36–61)	2.2 (1.6–2.5)	2.0 (1.7–2.4)	11 (7–27)	21 (17–22)	313 (209–355)	247 (178–419)	1213 (768–2717)	–		

*^a^*Stricture affecting common hepatic duct and first-order ducts bilaterally.

*^b^*Values are medians (interquartile range).

Asystolic time: time from asystole to in situ perfusion; . DBD, donation after brain death; DCD, donation after circulatory determined death; CIT, cold ischemic time; MEAF, model for early allograft function^14^; MRCP, magnetic resonance cholangiopancreatography; NA, not available; NESLiP, normothermic ex situ liver perfusion; notTx, not transplanted; Tx, transplanted; US DRI, United States Donor Risk Index^16^; UK DLI, United Kingdom Donor Liver Index^17^; withdrawal time, time from withdrawal of treatment to asystole.

**FIGURE 8.**
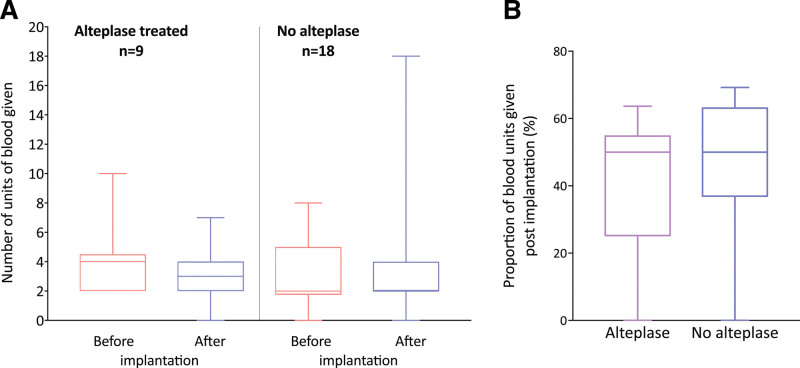
Intraoperative blood transfusion requirements of alteplase-treated and comparator livers. A, Number of packed red cell units given. This shows the number of units of packed red cells given before and after implantation in each case for livers treated with alteplase (n = 9) or not receiving alteplase (n = 18) during NESLiP. B, Proportion of packed red cell units given postimplantation as proportion of total blood units used. This figure shows the same data as above, but the number of units given postimplantation is given as a percentage of the total blood replacement during surgery. NESLiP, normothermic ex situ perfusion.

There was no difference in the blood transfusion requirements during transplantation of the 9 DCD livers undergoing NESLiP with alteplase when compared with the last 18 NESLiP livers that had not received alteplase (Figure 8). One alteplase-treated liver and 2 comparators underwent reexploration for bleeding on day 1. There were no biliary leaks or anastomotic strictures in treated livers.

### Histology of Nontransplanted Alteplase-treated Livers

Histology of nontransplanted alteplase-treated livers was reviewed. The extrahepatic bile ducts of most livers had varying degrees of stromal necrosis and epithelial loss, with loss of peribiliary glands. Patchy stromal necrosis was noted in some first-order ducts, but most appeared normal, as did higher-level ducts of all alteplase-treated livers. Peribiliary glands were variably seen in these livers with no evidence of pathological loss. The 3 livers of group A, which were perfused with alteplase alone without FFP (regimen A), showed epithelial loss and extensive stromal necrosis of intrahepatic ducts.

## DISCUSSION

Using D-dimers flushed out during NESLiP as a surrogate for the presence of fibrin within the vascular tree of donor livers, we have shown that both DBD and DCD livers contain a varying burden of fibrin, which is present before starting NESLiP and, for DBD livers at least, is related to the duration of cold ischemia. For DBD livers, the 2-h D-dimer concentrations during NESLiP also differentiated livers passing or failing viability assessment. High levels of D-dimers in all transplanted livers were associated with poor transplant outcomes and a higher incidence of biliary complications. Using alteplase with FFP, we demonstrated a greater release of D-dimers into the perfusate, suggesting a possible means to remove the accumulated fibrin.

Fibrin deposition in the sinusoids of livers undergoing NESLiP has been noted by others,^12,18^ although our interpretation of its presence differs from these previous reports with the additional insight gained from our effluent and alteplase data. Our study provides supporting evidence that fibrin microthrombi may have a causative role in the occurrence of bile duct complications, including NAS, in both DCD and DBD livers and shows, for the first time, an association with transplant outcome. Two of the 8 transplant recipient deaths were from multiorgan failure in association with venous outflow obstruction with a large transhepatic venous gradient; these livers had high levels of D-dimers and it is likely that fibrin deposition contributed to sinusoidal obstruction, a phenomenon that has been described elsewhere.^12,18^ Two deaths were from primary nonfunction in livers with high D-dimers transplanted into a relatively hostile surgical field after a large operative blood loss, a circumstance that challenges even the best liver. The third PNF in this series was in a liver that had been assessed on NESLiP after failing criteria during NRP; the underlying hepatocellular damage was underestimated with glycogen depletion and failure of glycolysis not appreciated during assessment.^19,20^

The correlation of D-dimer release with the duration of cold ischemia provides a possible explanation for the patchy appearance of livers perfused after prolonged ischemia and an additional explanation for the poorer outcome of such livers. The relationship between cold ischemia and D-dimer release did not hold for DCD livers. The confounding effects of additional periods of warm ischemia probably explain why there was no correlation between D-dimers and CIT with DCD livers. The observation of fibrin microthrombi in livers is similar to the demonstration of fibrin “microthrombi” accumulating within kidneys during cold ischemia^21-23^ and follows the original observations of Turunen et al,^24^ who showed that D-dimers can be flushed out of kidneys after cold storage.

Fibrinolytic treatment with alteplase and FFP caused more D-dimer release than nontreated NESLiP livers, suggesting a significant occult fibrin burden within livers of both donor types. More noteworthy are the livers that had 2-h D-dimer levels of 27 000 and 43 000 ng/mL and that have excellent graft function, despite these D-dimer levels being considerably higher than the values in the nonalteplase-treated highest quartile in which concentrations of 4000 to 16 000 ng/mL were associated with transplant failure (77% at 1 y) and NASs (38% at 1 y). The observation that most of the D-dimers are released in the first hour of NESLiP suggests that only preformed fibrin is being cleared rather than freshly deposited fibrin because the half-life of D-dimers in the human is between 5 and 15 h.

One of the livers receiving fibrinolytic treatment developed NAS, which involved the hilar ducts only. None of the other alteplase/FFP-treated livers developed anastomotic or NAS or leaks, and it was reassuring that there was no evidence of excess postimplant bleeding in the transplanted livers, based on the requirement for blood transfusion. Review of the extrahepatic duct histology of alteplase/FFP-treated livers showed stromal necrosis and epithelial loss in the common ducts of all livers, with half of the livers showing patchy stromal necrosis and epithelial loss affecting some of the higher ducts. We have also observed in our nonalteplase NESLiP that the pattern of NAS we see is dominated by proximal strictures. It is possible that this is related to trauma from the bile catheter placed at the start of NESLiP or that it reflects the difficulty in rapidly clearing small vessels of fibrin plugs in advance of infarction occurring during NESLiP. Another explanation, and one we favor, is that, in tying the bile catheter into the extrahepatic duct, the distal outflow from the periductal vessels is blocked, so the alteplase/FFP laden perfusate cannot penetrate adequately into that area. Our current practice is to delay bile duct cannulation for a period to allow these vessels to clear.

The observation that DBD livers that failed our viability criteria during NESLiP had significantly higher 2-h D-dimer levels than DBD livers that satisfied our viability criteria may give an insight into the pathology underlying poor function during NESLiP. Whether there is a causal relationship between poor perfusion parameters and preformed fibrin within the vasculature of donor livers cannot be confirmed in this study.

The absence of a difference in D-dimer levels between DCD livers that passed or failed viability criteria is less easy to explain, but the higher degree of selection of DCD livers based on bile chemistry may be partly responsible. More work needs to be done to explore the relationship between viability and fibrin load. Nevertheless, a strong case can be made for the inclusion of D-dimer levels when assessing the viability of all livers undergoing NESLiP, and bile duct viability in particular, if fibrinolytic treatment is not performed. In our recent article looking at factors that predict early graft function, we were able to account for only 30% of the variability in the model for early allograft function score by the currently favored biochemical variables, and we showed that our widely adopted bile chemistry criteria were also not able to robustly predict bile duct viability.^20^ We believe that the addition of D-dimers to the assessment of livers that have not been treated with fibrinolytics would increase the predictive ability of our criteria, and moreover, although the currently used criteria predict early graft function, D-dimer levels may be associated with longer-term transplant survival.

It was notable that the D-dimer levels of most of the DBD and DCD livers were higher than those of the NRP-treated livers. Although this might be explained by the heparinization during NRP or by hyperfibrinolysis during NRP, which has been reported in uncontrolled donors,^25^ it may be that NRP livers have experienced a preconditioning stimulus^26^ and are better able to tolerate ischemia and thus produce less ischemia-related fibrin. It is noteworthy that NRP has been associated with a very low incidence of cholangiopathy and improved transplant outcomes in national series, which would be explicable by the lack of intrahepatic fibrin we demonstrated.^3,4,27,28^ Superior renal function of NRP-recovered kidneys may also be explicable by the presence of fewer glomerular fibrin microthrombi, something that needs to be assessed formally.^29^

D-dimers are usually a consequence of plasmin degradation of clot, but other processes have been reported to cause D-dimer release in vivo,^30,31^ and we have shown here that simple flushing of a liver in the cold is sufficient to cause some release. In vivo, D-dimer levels have been related to clot burden in patients with deep vein thrombosis and pulmonary embolism, as well as being high during thrombolytic treatment.^31^ We noted the highest 2-h D-dimer levels in 2 recipients whose livers had undergone alteplase/FFP treatment, suggesting these livers had a high clot burden, although their postoperative course has been unremarkable probably as a consequence of that therapy. Unfortunately, it is not possible to estimate the clot burden in livers not undergoing fibrinolytic therapy, and our study is limited by the inability to relate the D-dimer levels either in the flush effluent or in the perfusate to clot burden.

HOPE has been associated with a low rate of NAS, although, in a recent randomized trial of dual HOPE (D-HOPE), the actual incidence of any NAS on magnetic resonance cholangiopancreatography was 60%, albeit only 6% were reported to be clinically significant.^32^ On the basis of our observations, one might speculate that the reduction in clinically significant NAS may simply reflect flushing out fibrin from the peribiliary vascular plexus before reperfusion in vivo. If this were the case, one might speculate that HOPE via the portal vein alone might be more efficient at retrograde clearance of fibrin microthrombi than D-HOPE, in which fibrin is flushed antegrade down the artery to impact in the small peribiliary capillaries. It would be interesting to assay the perfusate of HOPE/D-HOPE-treated livers and correlate D-dimer levels with the prevalence of clinically “significant” and “nonsignificant” biliary strictures. Anecdotally, we analyzed the 3-L D-HOPE perfusate of one 33-y-old DCD liver perfused for 2 h and found it contained 1686 ng/mL D-dimers (implying ~5.06 µg released in total), well above the median level for our simple 2-L effluent flush of 219 ng/mL (0.43 µg in total) and nearer the median 2-h nonalteplase NESLiP value of 2346 ng/mL (~3.05 µg D-dimer in total).

We chose alteplase as the plasminogen activator because of its short half-life of only a few minutes in vivo.^33^ This contrasts with streptokinase and urokinase, which have longer half-lives (around 20–30 min) and a slower onset of peak fibrinolytic activity.^34,35^ Alteplase is metabolized by the liver; hence, its half-life is also likely to be short during NESLiP, something that is supported by the lack of significant postimplantation bleeding.

In the absence of clinical-grade plasminogen, we opted to use FFP as a source of plasminogen for the alteplase to activate. The optimal fibrinolytic regimen with alteplase would appear to involve an initial bolus and an infusion of no more than an hour. We believe it is important to have an exogenous source of plasminogen—we have separately showed that very little plasminogen is actually produced in a cohort of NESLiP livers (unpublished data). It is notable that the FFP-free alteplase regimen was associated with more severe histological changes in intrahepatic ducts. The disadvantage of using FFP is the batch-to-batch variation in plasminogen content and also variable levels of alpha-2-antiplasmin and other plasmin inhibitors.

We delivered the alteplase and FFP as separate infusions either into the perfusate reservoir, where alteplase and FFP mixed, or directly into the hepatic artery cannula, where FFP and alteplase mixed at the point of entry into the cannula, the latter approach being used to avoid any loss of efficacy because of the rapid metabolism/neutralization of plasmin. It is not clear that there was a benefit of either strategy, and this is being further examined. Plasminogen is now available clinically for the treatment of plasminogen deficiency, and a nonclinical formulation was used in the only other published series of fibrinolysis of livers during NESLiP, although none of those livers were transplanted.^13^

In summary, our D-dimer data suggest that donor livers carry a significant burden of intravascular fibrin, and this affects viability assessment and causes a significant reduction in transplant survival and high rates of biliary complications. Fibrinolysis during NESLiP appears to be a promising way to address this phenomenon.

## ACKNOWLEDGMENTS

We are grateful to the organ donors and their families for permission to use donated livers in this research. We are also grateful to those patients who took part in the trials from which the samples studied were taken.

The perfusions took place in the main operating theaters of our hospital, and we are indebted to Gareth Hayman for facilitating this activity. We are also grateful to our colleagues, surgeons, hepatologists, and anesthetists, who have tolerated and supported our work.

## Supplementary Material




