# Supplementary figures and images for: D-dimer Release From Livers During Ex Situ Normothermic Perfusion and After In Situ Normothermic Regional Perfusion: Evidence for Occult Fibrin Burden Associated With Adverse Transplant Outcomes and Cholangiopathy
Source: Transplantation. 2023 May 23;107(6):1311–21. doi: 10.1097/TP.0000000000004475 (PMC10205116; doi:10.1097/TP.0000000000004475)

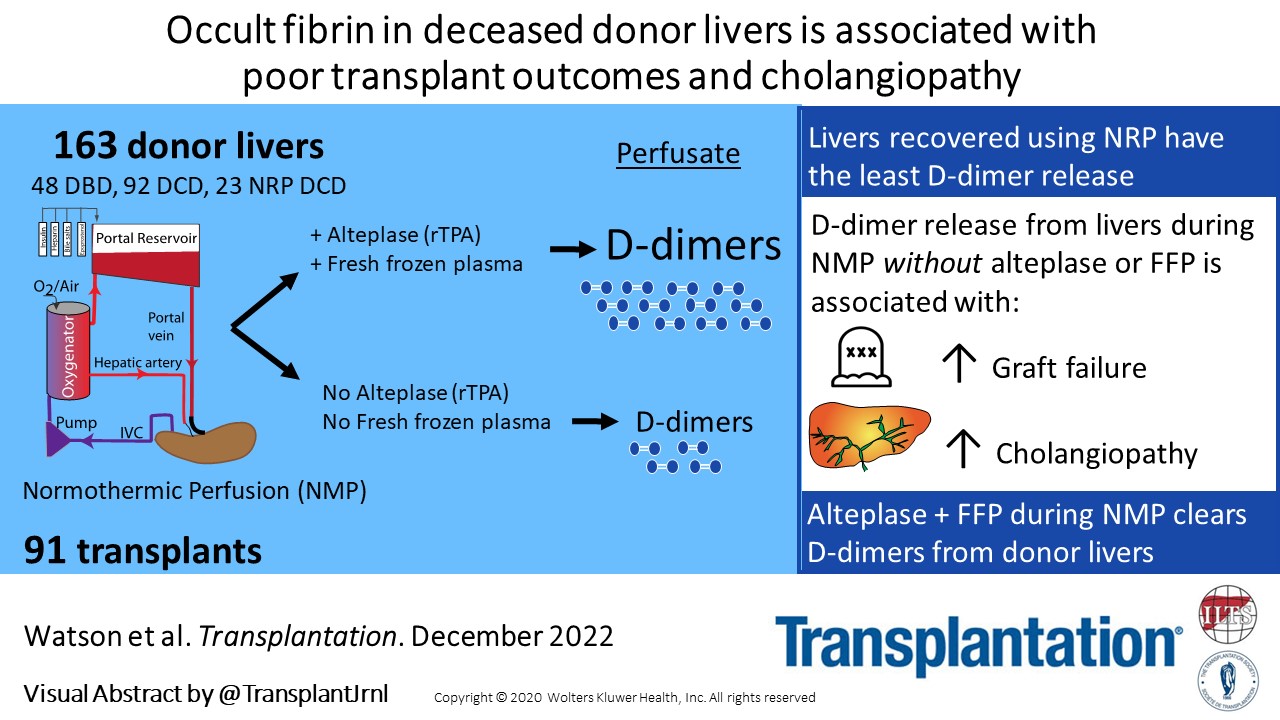

Supplement: Supplementary file 1 [file tpa-107-1311-s001.jpg]
