# Supplementary material for: D-dimer Release From Livers During Ex Situ Normothermic Perfusion and After In Situ Normothermic Regional Perfusion: Evidence for Occult Fibrin Burden Associated With Adverse Transplant Outcomes and Cholangiopathy
Source: Transplantation. 2023 May 23;107(6):1311–21. doi: 10.1097/TP.0000000000004475 (PMC10205116; doi:10.1097/TP.0000000000004475)

D-dimer release from livers during *ex situ* normothermic perfusion: evidence for occult fibrin burden associated with adverse transplant outcomes and cholangiopathy.

Watson et al.

SUPPLEMENTAL DIGITAL CONTENT

Table of contents.

Methods: Normothermic liver perfusion 2

Methods: D-dimer concentration measurement 2

Methods: Statistical analysis 3

Table S1. Causes of death and graft failure 4

Figure S1. 2-hour perfusate d-dimer concentrations for DBD and DCD livers that passed and failed viability criteria for transplantation. 5

Figure S2. D-dimer release after 2 hours of NESLiP in livers that had previously undergone NRP according to the indication for perfusion. 6

Figure S3. D-dimer concentrations at two hours comparing non-alteplase livers perfused with 5% Human Albumin Solution and Gelofusine 7

Figure S4. Correlation of 2 hour d-dimer perfusate concentrations during NESLiP with duration of cold ischaemia 8

Methods: Normothermic liver perfusion

Livers undergoing NESLiP in this study were perfused either using the Liver Assist (XVIVO, The Netherlands, n=29) or the *metra* (OrganOx, UK, n=134). The perfusate comprised a combination of packed red cells and either Gelofusine (BBraun, Germany) or 5% Human Albumin Solution (HAS), together with amino acids, calcium, magnesium, and heparin. During perfusion infusions of heparin, insulin, epoprostenol and, on the *metra* only, bile salts were given for the duration of perfusion. Perfusate samples were taken at regular time points to monitor the liver and samples frozen for future analysis.

Methods: D-dimer concentration measurement

D-dimers were measured using latex immunoturbidometry with the D-Dimer HS assay on the ACLTOP 750CTS analyser (Werfen, Barcelona, Spain) according to manufacturer's instructions and measured at 671nm . Briefly, polystyrene latex particles are coated with the Fab fragment of a monoclonal antibody directed against the D-Dimer domain. Bound D-Dimer in patient samples agglutinate latex particles, and a proportional decrease in light transmittance is measured using the automated coagulometers optical detection system. D-Dimer concentration, measured in ng/mL, is quantified from a stored calibration curve constructed with a D-Dimer standard (Werfen, Barcelona, Spain) serially diluted. This provides a linear range up to 3450 ng/mL. For results higher than the limit of the calibration curve, a further dilution of the patient plasma is performed to obtain transmittance values within the linear range of the assay. At this point the dilution factor is corrected for and the result obtained.

Methods: Statistical analysis

Statistical analyses were undertaken using Prism v7.0e (GraphPad Software Inc, San Diego USA) and SAS version 9.4 (SAS Institute Inc., NC, USA). Between group comparisons of two groups were undertaken using the Kolmogorov-Smirnov test, and the Kruskal-Wallis test for more than two groups. Survival comparisons were made using the logrank test.

Cox Proportional Hazard Regression models were used to model transplant survival (the time from transplant to either graft failure or patient death), censored at one year, as well as time from transplant to development of strictures, censored at one year. Both models were adjusted for donor age, cold ischaemia time, and donor type. Donor type was considered as a 3-level categorical variable – DBD, standard DCD, and NRP. D-dimer level was then added as a continuous variable to the models. D-dimer level was considered to significantly impact survival or stricture development if it reduced the model deviance significantly ($p<0.05$) according to the likelihood ratio test (LRT).

Table S1. Causes of death and graft failure

2-hour d-dimers (ng/ml)	2-hour d-dimer quartile	Days post op	Cause of death / graft failure
1381	1	9	Patient undergoing retransplant who lost graft due to fulminant liver failure secondary to acute antibody mediated rejection. Patient died the following day of multiorgan failure.
1431	2	2	Retransplanted for primary non function
2383	3	229	Retransplanted for cholangiopathy. Died day 306
3733	3	57	Died of multi-organ failure in the context of venous outflow obstruction. Venous gradient across liver of 12 to 17mmHg on day 23, increasing to 20mmHg by day 62 with 16mmHg gradient from hepatic vein to right atrium. Heterogeneous appearance of liver parenchyma on CT scan
3978	3	1	Died with primary non function in context of a heavy intraoperative blood loss
4422	4	95	Died from multi-organ failure and chest sepsis having had poor graft function post-transplant
5614	4	1	Died from primary non function in context of a heavy intraoperative blood loss
6320	4	45	Died from multi-organ failure in the context of venous outflow obstruction. Gradient of 13 to 20mHg across liver on day 25, with a gradient of 16mmHg from hepatic veins to right atrium.
7852	4	360	Retransplanted for hepatic artery thrombosis with cholangiopathy and hepatic abscesses.
16298	4	2	Died from cardiac failure with liver failure; had porto-pulmonary hypertension which was underestimated at assessment.

Figure S1. 2-hour perfusate d-dimer concentrations for DBD and DCD livers that passed and failed viability criteria for transplantation.

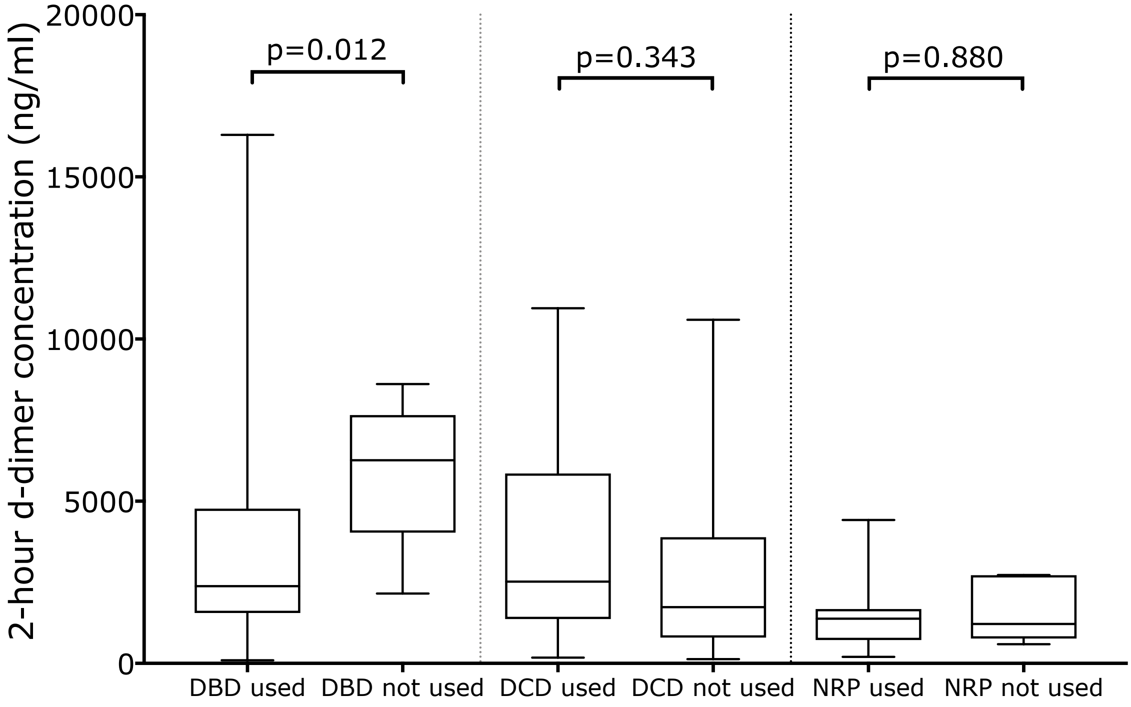

There is a significant difference in D-dimer levels for DBD livers that passed or failed viabiity criteria, which were based on hepatocyte function alone for DBDs. There was no difference in perfusate D-dimer concentrations for DCD livers passing or failing viability criteria, which, in additon to hepatocyte criteria, also were assessed according to bile chemistry.

Figure S2. D-dimer release after 2 hours of NESLiP in livers that had previously undergone NRP according to the indication for perfusion.

Each cross represents an individual liver. The horizontal bars represents the median, and interquartile ranges. The highest d-dimer concentration (11377 ng/ml) was in a liver which undergone thoraco-abdominal NRP during which thrombus was noted in the heart. The difference between the two groups was not significant (p=0.089), albeit the numbers are small.

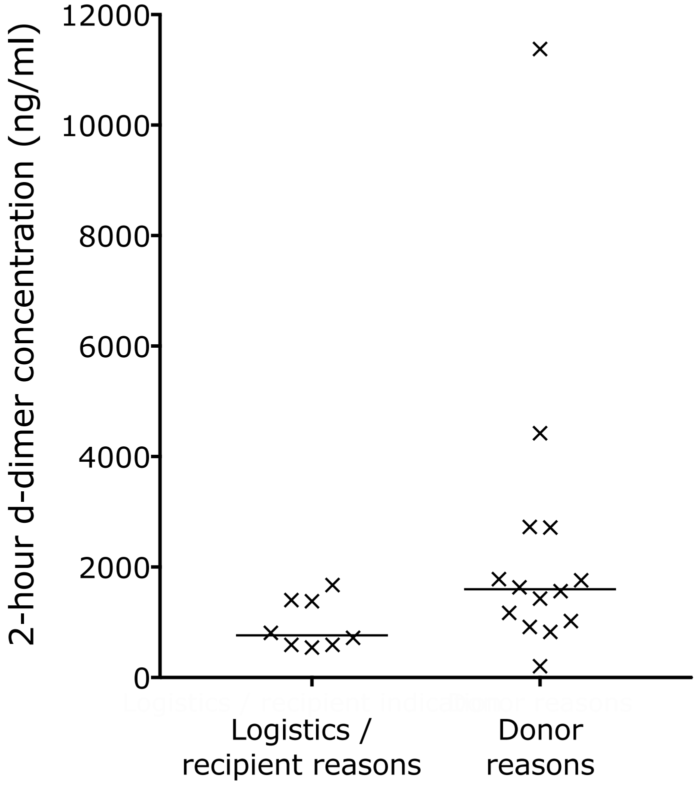

Figure S3. D-dimer concentrations at two hours comparing non-alteplase livers perfused with 5% Human Albumin Solution and Gelofusine

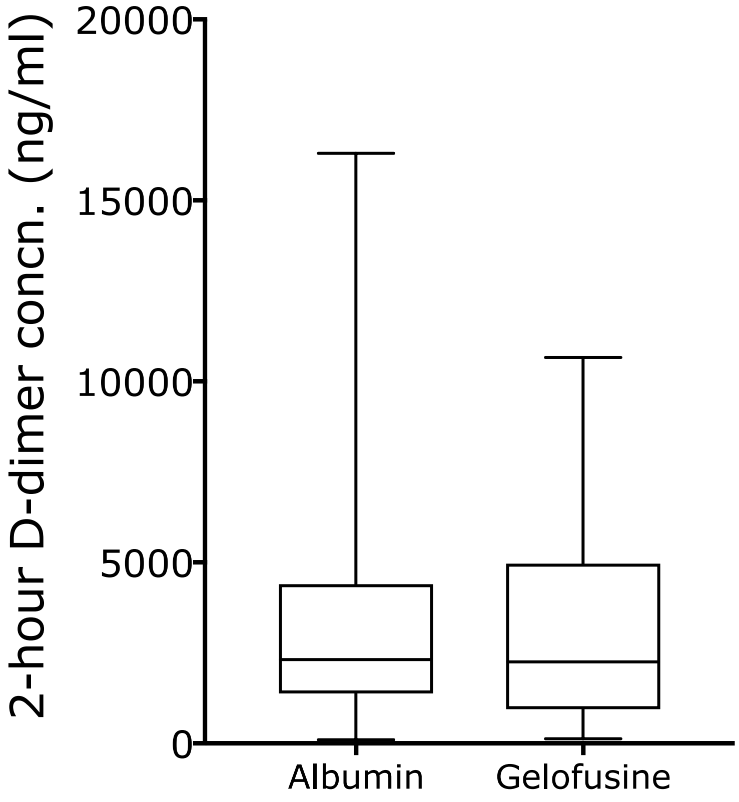

There is no significant difference between D-dimer release by perfusate (p=0.6704, Kolmogorov-Smirnov)

Figure S4. Correlation of 2 hour d-dimer perfusate concentrations during NESLiP with duration of cold ischaemia

a) During NESLiP for DBD donor livers

In interpreting the figure it should be borne in mind that the 2-hour d-dimer concentrations only reflect fibrin that has degraded, and not the total fibrin load within the liver.

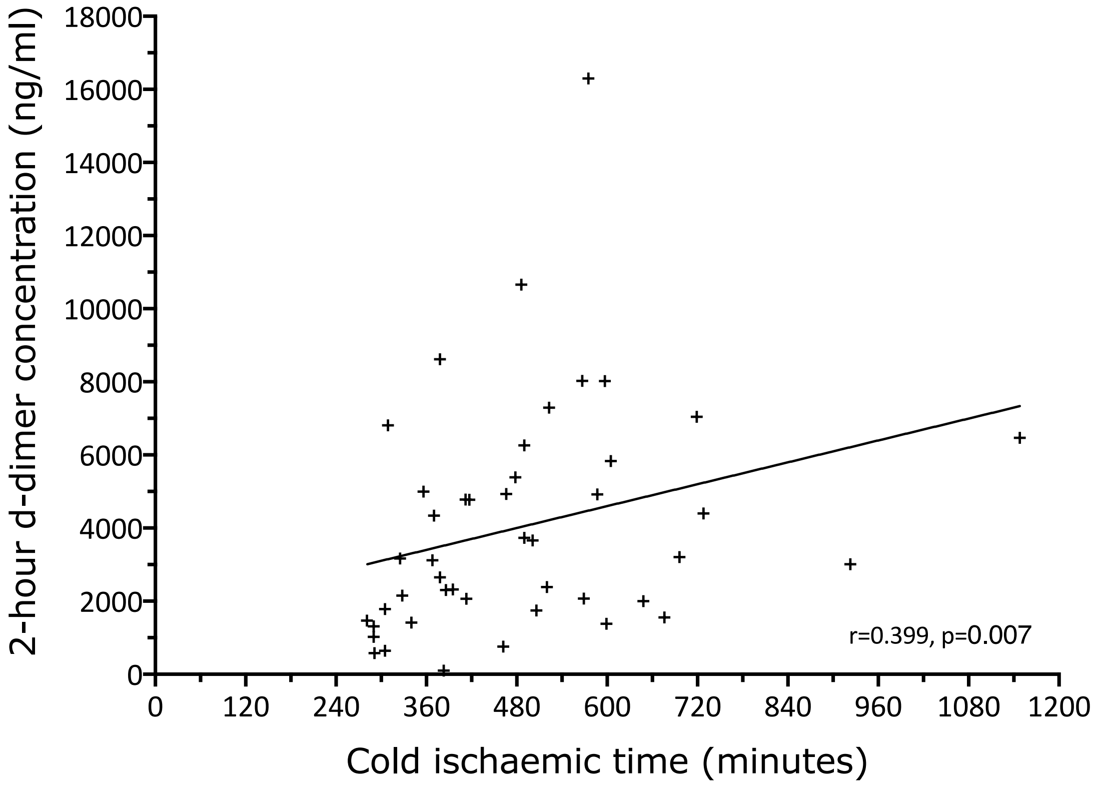

b) During NESLiP for DCD donor livers

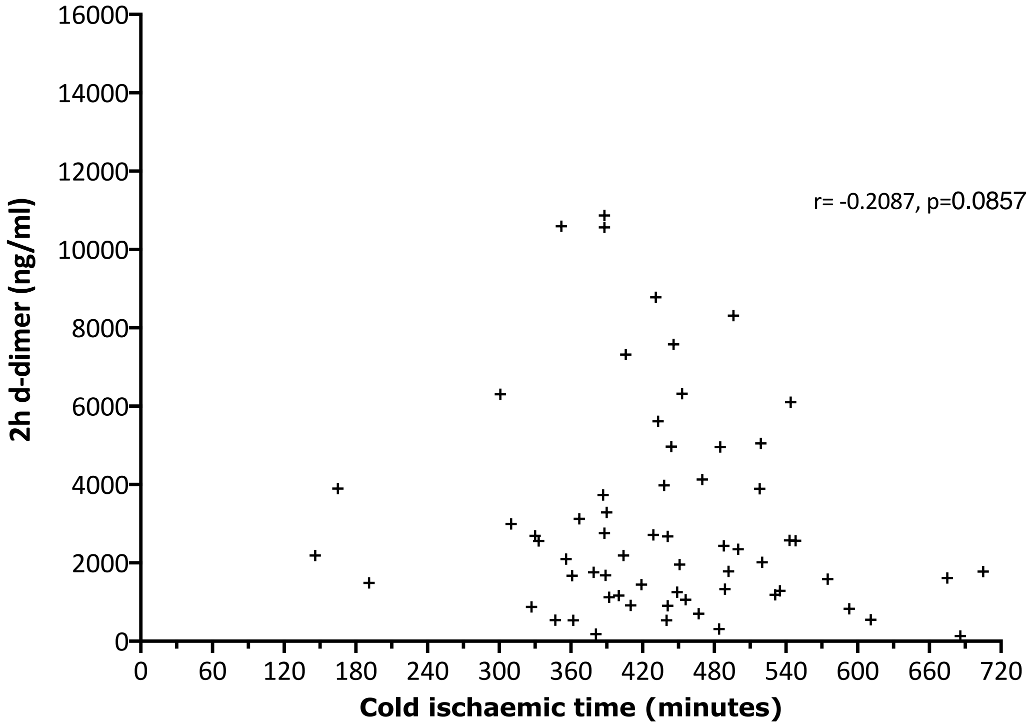

c) During NESLiP for DCD livers recovered using normothermic regional perfusion

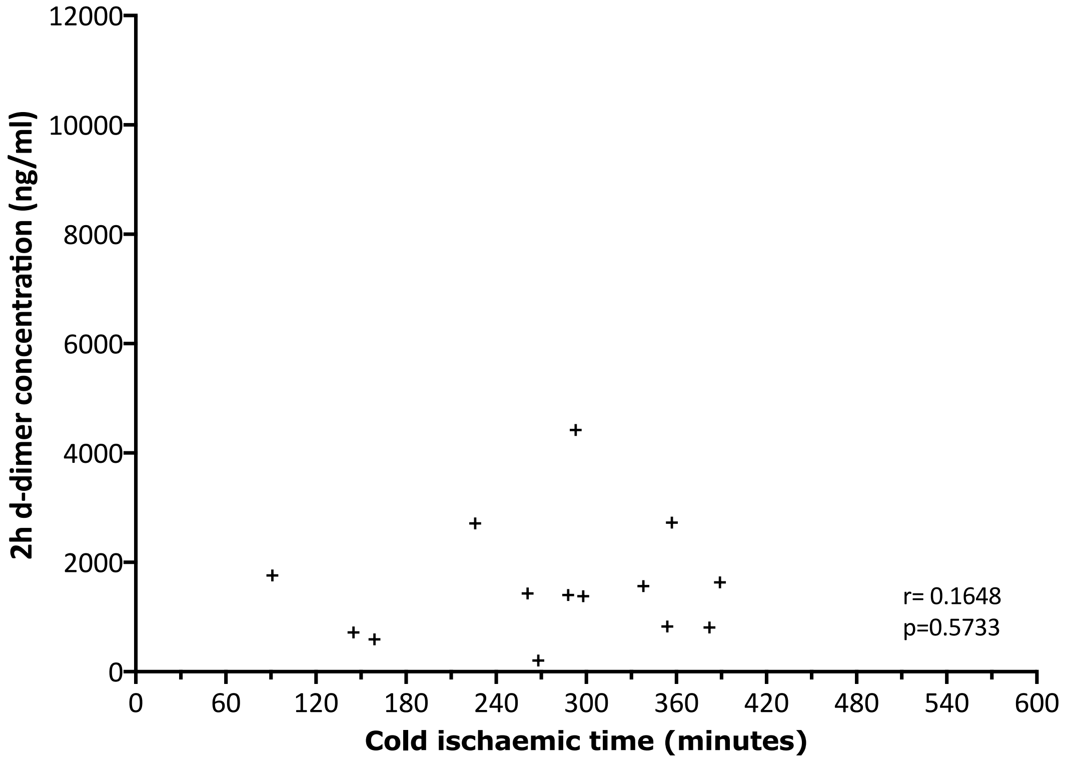

Supplement: Supplementary file 2 [file tpa-107-1311-s002.pdf]
